# Evolution of sodium-glucose co-transporter 2 inhibitors from a glucose-lowering drug to a pivotal therapeutic agent for cardio-renal-metabolic syndrome

**DOI:** 10.3389/fendo.2023.1111984

**Published:** 2023-01-30

**Authors:** Hiroki Akiyama, Akihiro Nishimura, Naru Morita, Toshitaka Yajima

**Affiliations:** ^1^ Medical Affairs, AstraZeneca K.K., Osaka, Japan; ^2^ Department of Internal Medicine, Urayasu Central Hospital, Chiba, Japan

**Keywords:** SGLT2 inhibitor, chronic heart failure, chronic kidney disease, cardiorenal protection, cardio-renal-metabolic syndrome

## Abstract

Cardio-renal-metabolic (CRM) syndrome, which involves type 2 diabetes mellitus (T2DM), chronic kidney disease (CKD), and heart failure (HF), is a serious healthcare issue globally, with high morbidity and mortality. The disorders that comprise CRM syndrome are independent can mutually affect and accelerate the exacerbation of each other, thereby substantially increasing the risk of mortality and impairing quality of life. To manage CRM syndrome by preventing vicious interactions among individual disorders, a holistic treatment approach that can simultaneously address multiple disorders underpinning CRM syndrome is of great importance. Sodium-glucose co-transporter 2 inhibitors (SGLT2i) lower blood glucose levels by inhibiting glucose reabsorption in the renal proximal tubule and were first indicated for the treatment of T2DM. Several cardiovascular outcome trials have demonstrated that SGLT2i not only lower blood glucose but also reduce the risk of hospitalization for HF and worsening renal function in patients with T2DM. Results have also suggested that the observed cardiorenal benefits of SGLT2i may be independent of their blood glucose-lowering effects. Several randomized controlled trials subsequently assessed the efficacy and safety of SGLT2i in patients without T2DM, and revealed considerable benefits of SGLT2i treatment against HF and CKD, regardless of the presence of T2DM. Thus, SGLT2i have become an essential therapeutic option to prevent the onset, slow the progression, and improve the prognosis of CRM syndrome. This review assesses the evolution of SGLT2i from a glucose-lowering drug to a therapeutic agent for CRM syndrome by evaluating epoch-making clinical studies, including randomized control trials and real-world studies.

## Introduction

It is well known that metabolic, cardiovascular (CV), and renal diseases closely interact with each other, forming the so-called cardio-renal-metabolic (CRM) syndrome (1, 2), where each disease is not an independent complication but instead affects and exacerbates the other disorders. The risk of developing CV diseases, including heart failure (HF), in patients with type 2 diabetes mellitus (T2DM) is approximately double that observed in individuals without T2DM (3). A considerable proportion of patients with T2DM (~50%) develop chronic kidney disease (CKD), referred to as diabetic kidney disease, a leading cause of end-stage renal disease (ESRD) (4–6). Moreover, decreased renal function is associated with an increased risk of the onset of HF and CV death (7–10). In fact, the prognosis of patients with concomitant T2DM, HF, and CKD is extremely poor (11). Given the vicious interactions among these disorders, holistic interventions that can simultaneously target the multiple disorders in CRM syndrome are of great importance (12).

One class of glucose-lowering drugs, sodium-glucose co-transporter-2 inhibitors (SGLT2i) (13), has recently attracted much attention owing to its cardiorenal benefits. Several CV outcome trials (CVOTs) have demonstrated that SGLT2i reduce the primary and secondary events associated with CV and renal diseases in patients with T2DM (14–16). In addition, several randomized controlled trials (RCTs) have subsequently shown significant efficacy of SGLT2i treatment against HF and CKD regardless of the presence of T2DM (17–21). Owing to these startling results, SGLT2i have evolved from a glucose-lowering drug to an essential therapeutic option to prevent the onset, slow the progression, and improve the prognosis of CRM syndrome. This review describes the evolutionary journey of SGLT2i with reference to epoch-making studies, including RCTs and real-world observational studies.

## Identification and development of SGLT2i

The development of SGLT2i started from the discovery of phlorizin in the bark of apple trees by Petersen in 1835 and the subsequent discovery of its effects on glucosuria and plasma glucose-lowering by von Mering in 1886 (22). In 1962, Alvarado and Crane showed that phlorizin competitively inhibits the cotransport of glucose and sodium in the proximal tubule of the kidney although the molecular entity of the transporter remained unknown (23). Two decades later, SGLT1, which transports D-glucose and D-galactose, was cloned from rabbit small intestine by Wright et al. at the University of California, Los Angeles (24). SGLT1 is predominantly expressed in S3 segments and assumed to be responsible for the reabsorption of 10% of the glucose filtered by the glomerulus (25). To elucidate the molecular entity of a similar glucose transporter in the proximal tubule responsible for the reabsorption of the remaining 90% of glucose, Kanai et al. at Osaka University screened a renal cDNA library using SGLT1 as a probe, which led to the identification of SGLT2 (26). In 1999, a Japanese pharmaceutical company developed T-1095, an undegradable SGLT2i. This is the first SGLT2i that was shown to enhance urinary glucose excretion and lower blood glucose levels in diabetic rats (27).

## SGLT2i for the prevention of diabetic complications

It is well known that patients with diabetes frequently develop micro- and macrovascular complications, which worsen the prognosis and quality of life (QOL) of patients. More recently, a multinational cohort study demonstrated that HF and CKD are early and frequent complications in patients with T2DM (10), and their risk of developing these complications is double that of individuals without T2DM (11). For managing diabetic complications, glucose-lowering drugs are of great importance, together with diet and exercise therapy. Because patients with diabetes require long-term treatment, glucose-lowering drugs must not only be effective in terms of reducing hyperglycemia but must also be safe in terms of the risks of hypoglycemia and vascular diseases. Despite these requirements, some glucose-lowering drugs were reported to be potentially associated with an increased risk of CV events (28, 29). This led the United States Food and Drug Administration (FDA) to issue guidance for the development of new antidiabetic drugs in 2008, mandating the so-called “CV no harm study” to evaluate the CV risks of newly launched drugs in longer-term clinical trials with prespecified CV endpoints, usually a composite primary endpoint of CV death, non-fatal stroke, and non-fatal myocardial infarction (MI) (three-point major adverse CV events; 3P-MACE) or 4P-MACE (3P-MACE plus unstable angina). The European Medicines Agency (EMA) also stated the need for CV no harm studies, with safety outcomes consisting of 3P-MACE or 4P-MACE, as well as other events such as revascularization and/or worsening of HF.

In consideration of the FDA and EMA guidance, a meta-analysis of 21 phase 2b/3 clinical trials was performed to evaluate the risk of an increase in CVD in studies performed for the regulatory approval of dapagliflozin. This meta-analysis revealed numerically lower incidences of the composite of 4P-MACE as well as the individual components (30, 31). Moreover, the meta-analysis revealed that, dapagliflozin significantly reduced hospitalization for HF (HHF), with a hazard ratio (HR) of 0.361 (95% confidence interval [CI], 0.156 to 0.838). Subsequently, a prospective CVOT for dapagliflozin, the DECLARE-TIMI 58 trial (*n* = 17,160; median follow-up, 4.2 years), was conducted (16, 32). Large CVOTs also evaluated the risk of CVD for other SGLT2i, namely, the EMPAREG-OUTCOME trial (*n* = 4,687; median follow-up, 3.1 years) and the CANVAS program (*n* = 10,142; median follow-up, 2.4 years) (14–16). There are some differences in the characteristics of patients included in these trials. One is the baseline CV risk. EMPA-REG OUTCOME enrolled patients with established CVD whereas the other trials enrolled patients with either established CVD or risk factors for CVD, resulting in a different prevalence of CVD at baseline (100% in EMPA-REG OUTCOME, 65.6% in CANVAS, and 40.6% in DECLARE-TIMI 58). Another major difference is baseline renal function. While EMPA-REG OUTCOME and CANVAS excluded patients with eGFR of <30 and ≤30 mL/min/1.73 m^2^, respectively, DECLARE-TIMI 58 excluded patients with a creatinine clearance of <60 mL/min. The percentage of patients with eGFR < 60 mL/min/1.73 m^2^ in each trial was 25.9% in EMPA-REG OUTCOME, 20.1% in CANVAS, and 7.4% in DECLARE-TIMI 58 (14, 33, 34). Despite these differences in patient characteristics, the three trials consistently showed non-inferiority for 3P-MACE versus placebo from the safety perspective. Furthermore, SGLT2i demonstrated superiority for 3P-MACE versus placebo in EMPA-REG OUTCOME and CANVAS but not in DECLARE-TIMI 58. This apparent difference may be explained by the lower baseline risk of CVD in patients enrolled in DECLARE-TIMI 58. Indeed, a prespecified subgroup analysis showed that dapagliflozin significantly reduced the relative risk of MACE by 16% in patients with previous MI (35). Other evidence supporting this notion is that the incidence rate of 3P-MACE was the lowest in DECLARE-TIMI 58 among the three CVOTs, indicating lowest statistical power in that study (36). Furthermore, a meta-analysis of these three trials demonstrated that SGLT2i reduced 3P-MACE by 11% compared to placebo with no heterogeneity (HR 0.89, 95% CI 0.83 to 0.96, p = 0.0014; Q statistic = 1.20, p = 0.55, I^2 =^ 0%) (37). For the individual components, SGLT2i reduced the risk of MI with no heterogeneity (HR 0.89, 95% CI 0.89 to 9.98, p = 0.0177; Q statistic = 0.03, p = 0.98; I^2 =^ 0%) and CV death with a significant intertrial difference (HR 0.84, 95%CI 0.75 to 0.94, p = 00023; Q statistic = 9.95, p = 0.0069; I^2 =^ 79.9%). Furthermore, there were significant risk reductions for CV death and all-cause mortality in EMPA-REG OUTCOME but not in the other two trials. The difference in baseline CV disease risk may explain these differing outcomes. The results of a meta-analysis showing a numerically higher incidence and a greater efficacy of SGLT2i in patients with a history of ASCVD than in patients with multiple risk factors support this notion (37).

In DECLARE-TIMI 58, non-inferiority of dapagliflozin to placebo with respect to 3P-MACE was first assessed and superiority was sequentially tested with hierarchical closed testing procedure. The study verified non-inferiority of dapagliflozin, but not superiority, as described above. In addition to 3P-MACE, the effects of SGLT2i were rigidly tested on a composite of HHF and CV death as a co-primary endpoint. DECLARE-TIMI 58 first demonstrated a statistically significant risk reduction in HHF/CV death, and EMPA-REG OUTCOME and CANVAS also reported positive effects on this outcome (14–16, 38, 39). Consistent with the results of a meta-analysis of phase 2b/3 trials, the CVOTs demonstrated that SGLT2i significantly reduced the incidence of HHF compared with placebo, with relative risk reductions ranging from 27% to 35% (**Table 1**). Additionally, two large RCTs, the CREDENCE trial and the VERTIS CV trial, showed beneficial effects of SGLT2i on HHF in patients with T2DM and CKD or established CVD (40, 41). Moreover, no heterogeneity among patients with and without a history of HF was reported in these five trials (**Table 1**). These results demonstrate the effects of SGLT2i on primary and secondary prevention of HHF in a broad range of patients with DM.

**Table 1:** Hospitalization for heart failure among patients with type 2 diabetes mellitus with and without a history of heart failure.

Trials	Events per 1,000 patient-years		HR (95% CI)
	Treatment	Placebo	
Overall population			
EMPA-REG OUTCOME (14)	9.4	14.5	0.65 (0.50 to 0.85)
CANVAS program (15)	5.5	8.7	0.67 (0.52 to 0.87)
DECLARE-TIMI 58 (16)	6.2	8.5	0.73 (0.61 to 0.88)
CREDENCE (40)	15.7	25.3	0.61 (0.47 to 0.80)
VERTIS CV (41)	7.3	10.5	0.70 (0.54 to 0.90)
History of HF			
EMPA-REG OUTCOME	40.7	52.4	0.75 (0.48 to 1.19)
CANVAS Program	14.1	28.1	0.51 (0.33 to 0.78)
DECLARE-TIMI 58	27.7	37.2	0.73 (0.55 to 0.96)
CREDENCE	39.3	48.9	0.76 (0.48 to 1.22)
VERTIS CV	16.9	26.2	0.63 (0.44 to 0.90)
No history of HF			
EMPA-REG OUTCOME	6.4	10.8	0.59 (0.43 to 0.82)
CANVAS Program	4.3	5.7	0.79 (0.57 to 1.09)
DECLARE-TIMI 58	4.0	5.6	0.73 (0.58 to 0.92)
CREDENCE	11.7	21.5	0.54 (0.39 to 0.75)
VERTIS CV	4.7	6.0	0.79 (0.54 to 1.15)

HR, hazard ratio; CI, confidence interval; HF, heart failure.

The aforementioned large RCTs also evaluated the effects of SGLT2i on renal function. For example, dapagliflozin reduced the risk of renal events, defined as a composite of a sustained eGFR decrease by ≥40% to an eGFR of <60 mL/min/1.73 m^2^, ESRD, and renal death and it slowed the rate of eGFR decline (34). In addition, dapagliflozin was associated with less deterioration and more amelioration of albuminuria compared with placebo (42). These renoprotective effects were robustly examined in CREDENCE, which enrolled 4,401 patients with T2DM and CKD (40). Canagliflozin reduced the risk of a renal-specific composite of ESRD, doubling of the creatinine level, or death from renal causes, a secondary endpoint specified in a sequential hierarchical testing procedure. Furthermore, similar to HF, this renoprotective efficacy observed in large RCTs was consistent regardless of renal function at baseline, suggesting the prevention of onset and progression of CKD by SGLT2i (**Table 2**) (34).

**Table 2:** Renal composite outcomes (worsening eGFR, end-stage renal disease, or renal death) according to renal function among patients with type 2 diabetes.

Trial	Events per 1,000 patient-years		HR (95% CI)
	Treatment	Placebo	
Overall population			
EMPA-REG OUTCOME (14)	6.3	11.5	0.54 (0.40 to 0.75)
CANVAS Program (15)	5.5	9.0	0.60 (0.47 to 0.77)
DECLARE-TIMI 58 (16)	3.7	7.0	0.53 (0.43 to 0.66)
CREDENCE (40)	27.0	40.4	0.66 (0.53 to 0.81)
VERTIS CV (43)	9.3	11.5	0.81 (0.63 to 1.04)
eGFR <60 mL/min/1.73 m^2^			
EMPA-REG OUTCOME	NA	NA	0.66 (0.41 to 1.07)
CANVAS Program	11.4	15.1	0.74 (0.48 to 1.15)
DECLARE-TIMI 58	8.9	15.2	0.60 (0.35 to 1.02)
CREDENCE (45 to <60)	33.4	63.1	0.52 (0.38 to 0.72)
VERTIS CV	16.3	14.7	0.90 (0.59 to 1.38)
eGFR 60 to <90 mL/min/1.73 m^2^			
EMPA-REG OUTCOME	NA	NA	0.61 (0.37 to 1.03)
CANVAS Program	4.6	7.4	0.58 (0.41 to 0.84)
DECLARE-TIMI 58	4.2	7.8	0.54 (0.40 to 0.73)
CREDENCE	14.9	18.5	0.81 (0.52 to 1.26)
VERTIS CV	10.5	7.0	0.66 (0.46 to 0.94)
eGFR ≥90 mL/min/1.73 m^2^			
EMPA-REG OUTCOME	NA	NA	0.21 (0.09 to 0.53)
CANVAS Program	3.8	8.1	0.44 (0.25 to 0.78)
DECLARE-TIMI 58	2.5	4.9	0.50 (0.34 to 0.73)
CREDENCE	NA	NA	NA
VERTIS CV	9.3	9.6	1.04 (0.63 to 1.73)

eGFR, estimated glomerular filtration rate; HR, hazard ratio; CI, confidence interval. NA, Not available.

In addition to these RCTs, real-world studies have investigated the cardiorenal benefits of SGLT2i in clinical settings (44–50). The CVD-REAL 2 trial, using data from six countries, showed a significant reduction in HHF, MI, stroke and all-cause mortality compared with other oral glucose-lowering drugs regardless of the history of CVD in patients with T2DM (44). A follow-up study of CVD-REAL 2 using data from 13 countries over a longer period yielded similar results when comparing SGLT2i with dipeptidyl peptidase-4 inhibitors (DPP-4i) (49). Several other real-world studies consistently reported a reduced risk of CV events and death with SGLT2i compared with other glucose-lowering drugs or DPP-4i (46, 47, 51).

In addition to these CV benefits, the renoprotective effects of SGLT2i have been demonstrated in real-world settings. The CVD-REAL 3 trial, a multinational observational cohort study, demonstrated a significant difference in the mean annual rate of change in eGFR between SGLT2i (0.46 mL/min/1.73 m^2^ per year; 95% CI, 0.34 to 0.58) and other glucose-lowering drugs (−1.21 mL/min/1.73 m^2^ per year; 95% CI, −1.35 to −1.06) (45). SGLT2i were also shown to slow the decline in eGFR in patients with T2DM and CKD in a Japanese real-world study (50). Moreover, SGLT2i can reduce the risk of ESRD (45). Although these real-world studies utilized propensity-score matching, the results should be interpreted carefully due to the possible involvement of unconsidered confounders. These lines of real-world evidence can contribute to complement the results of RCTs and demonstrate the cardiorenal benefit of SGLT2i in patients with T2DM in clinical settings.

The real-world studies comparing SGLT2i and other glucose-lowering drugs suggest that the cardiorenal benefit seems to be independent of glucose-lowering effect. This idea was tested in a secondary analysis of clinical trials in which canagliflozin was compared with glimepiride (52). Despite the similar level of reductions in HbA1c, the decrease in systolic blood pressure was larger in the canagliflozin group than in the glimepiride group. Furthermore, the eGFR decline in the canagliflozin group was significantly slower than that in the glimepiride group. Another clinical trial enrolling Japanese patients with T2DM and CKD demonstrated that neither baseline HbA1c nor the changes in HbA1c significantly affected the decrease in the urinary albumin-to-creatinine ratio observed during 24 weeks of dapagliflozin treatment (53). Because SGLT2i lower blood glucose level by promoting the excretion of glucose into urine, their glucose-lowering effects become weaker as eGFR declines (54). Nonetheless, CVOTs reported that SGLT2i consistently reduced the risk of CV and renal outcomes even in patients with lower eGFR (≤45 mL/min/1.73m^2^) (40, 55). Collectively, these findings suggest that SGLT2i confer a cardiorenal benefit independently of their glycemic effects.

The 2022 American Diabetes Association (ADA) standards of medical care in diabetes recommend a comprehensive approach to reduce the risk of diabetic complications, in which the following four factors are considered to be fundamental: management of glycemia, blood pressure, and lipids and the incorporation of specific therapies with efficacy on CV and renal outcomes (56). The importance of multifactorial interventions to target glycemia, blood pressure, and lipids is well established (57–59). Based on the evidence provided by RCTs, SGLT2i and glucagon-like peptide-1 receptor agonists (GLP-1 RA) are recommended to reduce the risk of adverse CV and renal events. The 2021 European Society of Cardiology (ESC) guidelines on CV disease prevention in clinical practice recommend SGLT2i and GLP-1 RA for reducing CV and/or cardiorenal outcomes in patients with T2DM (60). From the viewpoint of HF, SGLT2i are recommended for patients with stage A HF. The ADA consensus report for HF recommends that SGLT2i are an expected element of care in all individuals with diabetes and symptomatic HF and should be used in individuals with high CV risk, including those with stage B HF (61). However, DPP-4i and thiazolidines are not recommended for patients with diabetes with stages B, C, and D HF. For all patients with T2DM and stage ≥3 CKD, the use of SGLT2i is strongly recommended in the 2022 ADA standards of medical care in diabetes owing to their effects on slowing CKD progression and reducing HF risk (62). This recommendation is also based on the finding that the renoprotective effects of SGLT2i were not dependent on eGFR despite an attenuated glucose-lowering effect in patients with an eGFR of <45 mL/min/1.73 m^2^ (**Table 2**).

## SGLT2i for the treatment of HF

HF is a diabetic complication that must be managed; simultaneously, it is a serious issue for individuals without diabetes. The global prevalence of HF is estimated to be approximately 64 million, and it is the world’s leading cause of hospitalization with a high risk of re-hospitalization (63, 64). As mentioned above, large CVOTs for SGLT2i have shown substantial risk reduction in HHF among patients with T2DM, including in patients with a history of HF (**Table 2**). The important remaining question is whether the beneficial effect of SGLT2i on HF can be extended to patients without T2DM.

The DAPA-HF trial was the first clinical trial to provide an answer to this question. The trial examined the efficacy and safety of dapagliflozin in addition to standard of care in patients with chronic HF and reduced ejection fraction (HFrEF) with or without DM (*n* = 4,744; median follow-up, 18.2 months) (17). Dapagliflozin achieved a 26% relative risk reduction for the primary outcome, defined as a composite of death from CV causes, HHF, and urgent HF visits, regardless of DM. Furthermore, this reduction was rapidly apparent, with a sustained statistically significant efficacy by 28 days after randomization (HR at 28 days 0.51, 95% CI 0.28 to 0.94) (65). Significant risk reduction was also observed for individual components of the composite outcome, with HRs of 0.82 (95% CI 0.69 to 0.98) for the risk of death from CV causes and 0.70 (95% CI 0.59 to 0.83) for the risk of HHF. Dapagliflozin was also associated with lower risk of death with a HR of 0.83 (95% CI 0.71 to 0.97) for death from any cause, and a HR of 0.79 (95% CI 0.63 to 0.99) for a composite of arrhythmia, resuscitated cardiac arrest and sudden death (66). In addition to these hard outcomes, DAPA-HF assessed QOL and showed a significant increase in the Kansas City cardiomyopathy questionnaire (KCCQ) score in the dapagliflozin group compared with the placebo group, indicating an improvement in patient-assessed symptoms. Although there were numerically fewer events in the dapagliflozin group than in the placebo group, there was no statistically significant difference in the renal composite outcome, defined as a ≥50% sustained decline in eGFR, ESRD, or renal death (HR 0.71, 95% CI 0.44 to 1.66). Consistently, the EMPEROR-Reduced trial, another large RCT of patients with HFrEF with or without DM, reported a 25% relative risk reduction in a composite of death from CV causes and HHF in patients treated with the SGLT2i empagliflozin (18). In addition, empagliflozin reduced the incidence of the composite renal outcome, defined as chronic dialysis, renal transplantation, a sustained reduction in eGFR of ≥40%, or sustained eGFR of <15 mL/min/1.73 m^2^ among patients with a baseline eGFR of ≥30 mL/min/1.73 m^2^ or <10 mL/min/1.73 m^2^ among patients with a baseline eGFR of <20 mL/min/1.73 m^2^. Empagliflozin also slowed the annual rate of decline in eGFR compared with placebo. A significantly greater improvement in the KCCQ score was reported in the empagliflozin group. Although EMPEROR-Reduced did not show a significant risk reduction for death from CV causes (HR 0.92, 95% CI 0.75 to 1.12) or death from any cause (HR 0.92, 95% CI 0.77 to 1.10), a meta-analysis of DAPA-HF and EMPEROR-Reduced showed significant risk reductions for both outcomes (67). These findings collectively show a cardiorenal benefit of SGLT2i in improving the prognosis and QOL of patients with HFrEF.

Approximately half of the patients with HF have LVEF of >40%, HF with preserved or mildly reduced ejection fraction (HFpEF or HFmrEF) (68, 69). The prognosis of this population is equivalently poor compared with HFrEF, with a 5-year mortality rate of approximately 40% (70). Several drugs with demonstrated benefits in HFrEF have been evaluated in HFmrEF and HFpEF, but none have shown conclusive efficacy (71–74). The EMPEROR-Preserved trial, which enrolled 5,988 patients with HFmrEF and HFpEF regardless of DM, evaluated the efficacy and safety of empagliflozin, and reported for the first time a positive result of a 21% relative risk reduction in a composite of CV death and HHF (HR 0.79, 95% CI 0.69 to 0.90) (20). This was accompanied by sustained, statistically significant efficacy by 18 days after randomization (HR at 18 days 0.41, 95% CI 0.17 to 0.99) (75). In that trial, empagliflozin significantly slowed the slope of the eGFR decline and relieved the patient’s symptoms and physical limitations, as measured by KCCQ (20, 76).

Although the results reported in EMPEROR-Preserved were epoch-making, the study left some uncertainties to be addressed. In particular, it was unclear whether these benefits are conserved in patients with a higher LVEF spectrum (>60%), in patients who start treatment during the subacute phase (i.e. during or soon after hospitalization), or in patients with a prior LVEF of ≤40% (HFrEF) that has since improved to >40% (HF with improved EF, HFimpEF). These gaps in evidence were addressed in the DELIVER trial, which evaluated the efficacy and safety of dapagliflozin in patients with HFmrEF and HFpEF (*n* = 6,263; median follow-up, 2.3 years) (21). In this trial, dapagliflozin significantly reduced the risk of the primary outcome events, CV death or worsening HF events, compared with placebo in the overall population (HR 0.82, 95% CI 0.73 to 0.92) and in a subpopulation of patients with an LVEF of <60% (HR 0.83, 95% CI 0.73 to 0.95). Consistent with the results of EMPEROR-Preserved, an early benefit of dapagliflozin was observed because the risk reduction for the primary outcome was statistically significant at 13 days after randomization and statistical significance was sustained from 15 days onward (77). An improvement in the KCCQ score was also reported, showing that dapagliflozin can ameliorate symptoms in patients with HFmrEF and HFpEF (21). Notably, DELIVER extended the findings of EMPEROR-Preserved trial based on the results of subgroup analyses, which demonstrated a consistent efficacy among patients with an LVEF of ≥60% (HR 0.78, 95% CI 0.62 to 0.98), HFimpEF (HR 0.74, 95% CI 0.56 to 0.94), and in the subacute phase (HR 0.78, 95% CI 0.6 to 1.03). Following evidence from a meta-analysis of DELIVER and EMPEROR-Preserved trials showing a consistent risk reduction for the composite of CV death and HHF (78), SGLT2i have been identified as the first drug that can be effective in patients with HFmrEF and HFpEF regardless of the presence of T2DM.

Four RCTs, DAPA-HF, DELIVER, EMPEROR-Reduced, and EMPEROR-Preserved, consistently showed the efficacy of SGLT2i in patients with HFrEF, HFmrEF, and HFpEF (**Table 3**). In addition, the SOLOIST-WHF trial assessed the efficacy and safety of sotagliflozin, a combined SGLT1 and SGLT2 inhibitor, in 1,222 patients with HF and T2DM, and demonstrated a significant risk reduction in a composite of CV death and worsening HF regardless of LVEF (>50% or ≤50%) (79) (**Table 3**). These results suggest that SGLT2i can be effective in patients with HF regardless of LVEF. This is a remarkable difference from the other drugs used for HF because the vast majority of them are only used to treat HFrEF owing to their attenuated effects in the higher LVEF spectrum (80). Two pooled meta-analyses of empagliflozin (pooled EMPEROR-Reduced and EMPEROR-Preserved, *n* = 9,718) and dapagliflozin (pooled DAPA-HF and DELIVER, *n* = 11,007) further support this notion (81, 82). Significant risk reductions for a composite of CV death and HHF were reported in both pooled analyses without heterogeneity across the full LVEF spectrum.

**Table 3:** Randomized controlled trials of patients with heart failure.

	DAPA-HF (17)	DELIVER (21)	EMPEROR-Reduced (18)	EMPEROR-Preserved (20)	SOLOIST-WHF (79)
Drug	Dapagliflozin	Dapagliflozin	Empagliflozin	Empagliflozin	Sotagliflozin
No. of patients	4,744	6,263	3,730	5,988	1,222
LVEF (mean, %)	31.1	54.2	27.2	54.3	35
Median NT-proBNP (median, pg/mL)	1,437	1,011	1,910	994	1,800
eGFR (mean, mL/min/1.73 m^2^)	65.8	61.0	62.2	60.6	49.7
T2DM (%)	45.1	44.8	49.8	49.1	100
Outcomes, HR (95% CI)					
HHF or CV death	0.74 (0.65 to 0.85)	0.82 (0.73 to 0.92)	0.75 (0.65 to 0.86)	0.79 (0.69 to 0.90)	0.67 (0.52 to 0.85)
HHF	0.7 (0.59 to 0.83)	0.77 (0.67 to 0.89)	0.69 (0.59 to 0.81)	0.71 (0.60 to 0.83)	0.64 (0.49 to 0.83)
CV death	0.82 (0.69 to 0.98)	0.88 (0.74 to 1.05)	0.92 (0.75 to 1.12)	0.91 (0.76 to 1.09)	0.84 (0.58 to 1.22)

LVEF, left ventricular ejection fraction; NT-proBNP, N-terminal pro–B-type natriuretic peptide; eGFR, estimated glomerular filtration rate; T2DM, type 2 diabetes mellitus; HR, hazard ratio; CI, confidence interval; HHF, hospitalization for heart failure; CV, cardiovascular.

A meta-analysis of RCTs that enrolled >1,000 patients with HF across the full LVEF spectrum provided a combined view of the effects of SGLT2i on mortality (78). For CV death and all-cause death, the overall HR was 0.87 (95% CI, 0.79 to 0.95) and 0.92 (95% CI, 0.86 to 0.99), respectively, without heterogeneity among trials. For dapagliflozin, the pooled analysis was prespecified to assess its effect on CV death and all-cause mortality because of insufficient statistical power for evaluating these hard outcomes in individual trials (82). In the overall HF population, a significant benefit of dapagliflozin on mortality was observed irrespective of LVEF, with a HR of 0.86 (95% CI 0.76 to 0.97) for CV death and a HR of 0.90 (95% CI 0.82 to 0.99) for all-cause death. The meta-analysis of 21,947 patients, including outpatients and hospitalized patients, also showed that SGLT2i significantly reduced worsening HF events and improved symptoms. Collectively, SGLT2i demonstrated efficacy in reducing mortality, managing HF, and improving QOL, and prompted us to consider SGLT2i as a fundamental therapy for a broad range of patients with HF.

The 2021 ESC HF guidelines recommend dapagliflozin/empagliflozin as one of the four cornerstone drug therapies, alongside angiotensin receptor neprilysin inhibitors (ARNI)/angiotensin converting enzyme inhibitors (ACEi), β-blockers (BB), and mineralocorticoid receptor antagonists (MRA) for reducing HHF and death in all patients with HFrEF (Class I) (83). Moreover, the 2022 American Heart Association (AHA)/American College of Cardiology (ACC)/Heart Failure Society of America (HFSA) recommend guideline-directed medical therapy for Stage C or D HFrEF, consisting of four medication classes, including SGLT2i, ARNI/ACEi/angiotensin receptor blocker (ARB), BB, and MRA (Class I) (84). Additionally, the Japanese Circulation Society (JCS)/Japanese Heart Failure Society (JHFS) 2021 guideline focused update on diagnosis and treatment of acute and chronic heart failure recommends SGLT2i regardless of the presence of diabetes to further decrease the risk of exacerbation of HF or CV death in patients with symptomatic HFrEF combined with optimal basic treatment (ACEi/ARB, BB, and MRA) (85).

SGLT2i were included in the 2022 AHA/ACC/HFSA HF guidelines for the treatment of HFmrEF and HFpEF to reduce HHF and CV mortality (Class 2a) (84). This is a higher recommendation than those allocated to ARNI/ACEi/ARB, BBs, and MRAs for the treatment of HFmrEF and HFpEF. The results of DELIVER are expected to be reflected in future guidelines and provide further guidance for the use of SGLT2i in clinical practice independently of EF. A meta-analysis of five RCTs, including DAPA-HF, DELIVER, EMPEROR-Reduced, EMPEROR-Preserved and SOLOIST-WHF (78), will likewise help to reinforce the guideline recommendations for the use of SGLT2i in patients with HFrEF, HFmrEF, and HFpEF, as well as the clinical need to initiate guideline-directed medical therapy to improve the outcomes of patients with HF.

## SGLT2i for the treatment of CKD

Although it was not a primary endpoint, protective effects of SGLT2i on renal function were demonstrated in three CVOTs, EMPA-REG OUTCOME, CANVAS, and DECLARE-TIMI 58, in which most enrolled patients had preserved renal function (14–16). The CREDENCE and DELIGHT trials further extended the renoprotective effects of SGLT2i to patients with T2DM and impaired renal function, in whom the glucose-lowering effects of SGLT2i are attenuated (40, 86). The proposed mechanism to explain these effects of SGLT2i on renal function, including reducing intrarenal hypoxia, may be relevant to patients with CKD without diabetes. The DAPA-CKD trial first evaluated the efficacy and safety of dapagliflozin in patients with CKD in the presence or absence of T2DM (*n* = 4,304; median follow-up, 2.4 years) (19). This trial was stopped early because of overwhelming efficacy. The primary outcome, assessed in terms of the time to the first event, was a composite of a sustained decline in eGFR of ≥50%, ESRD, and death from renal or CV causes. Dapagliflozin showed significant efficacy in reducing the risk of this composite by 39%, with a HR of 0.61 (95% CI 0.51 to 0.72), regardless of the presence of T2DM. Furthermore, there was no heterogeneity in the effects among the causes of CKD (87). Favorable effects of dapagliflozin were observed for each component of the composite outcome (19). In addition, the rate of eGFR decline was slower in the dapagliflozin group than in the placebo group (total eGFR slope difference 0.95 mL/min/1.73 m^2^, 95% CI 0.63 to 1.27).

DAPA-CKD also assessed the effects of dapagliflozin on HF events and mortality. The HR for the CV composite of death from CV causes or HHF was 0.71 (95% CI 0.55 to 0.92) and the HR for death from any cause was 0.69 (95% CI 0.53 to 0.88) (19). These benefits were consistent with those observed in patients with HF as described above. With the evidence for T2DM, these findings further emphasize the beneficial effects of dapagliflozin in patients with CRM syndrome.

Results of the EMPA-KIDNEY trial (*n* = 6,609; median follow-up, 2.0 years) were recently published (88). This trial was also stopped early because of the clear efficacy of empagliflozin, as previously shown in CREDENCE and DAPA-CKD. Empagliflozin significantly reduced the risk of the primary composite outcome, progression of kidney disease (initiation of maintenance dialysis or receipt of a kidney transplant, a sustained decrease in the eGFR to <10 mL/min/1.73 m^2^, a sustained decrease in eGFR by ≥40% from baseline, or death from renal causes) or CV death, by 28% (HR 0.72, 95%CI 0.64 to 0.82) regardless of diabetes status. The HRs for each component were 0.71 (95% CI 0.62 to 0.81) for progression of kidney disease and 0.84 (95% CI 0.60 to 1.19) for CV death. Furthermore, the rate of annual decline in eGFR was slower in the empagliflozin group than in the placebo group, with a between-group difference for the change from randomization to the final follow-up visit of 0.75 mL/min/1.73 m^2^ per year (95% CI 0.54 to 0.96). Overall, EMPA-KIDNEY confirmed the efficacy of SGLT2i in patients with CKD regardless of T2DM.

The KDIGO 2022 clinical practice guideline for diabetes management in CKD (89) is a focused update of the 2020 guidelines with a relatively short interval to include some recently published evidence, particularly RCTs of SGLT2i (17–20, 79). In the focused update, SGLT2i are recommended as first-line therapy for patients with T2DM and CKD, regardless of the level of glycemia, to improve their renal and CV outcomes. Based on recently published RCTs, the 2022 guideline advocates initiating SGLT2i for patients with T2DM and CKD and an eGFR of ≥20 mL/min/1.73 m^2^ instead of ≥30 mL/min/1.73 m^2^, and continuing SGLT2i treatment for as long as tolerated, even if eGFR decreases to <20 mL/min/1.73 m^2^, until kidney replacement therapy is initiated. SGLT2i are also recommended regardless of the patient’s level of albuminuria. Therefore, SGLT2i are considered a foundation of pharmacologic therapy for T2DM and CKD.

## Safety considerations

The safety profiles of SGLT2i as antidiabetic drugs were rigorously assessed in the large-scale CVOTs. Although the event rates were low, there was a consistent increased risk of diabetic ketoacidosis (DKA) in the SGLT2i group than in the placebo group in a meta-analysis that included EMPA-REG OUTCOME, CANVAS, and DECLARE-TIMI 58 (37). On the other hand, an increased risk of amputations and fractures was only observed in one trial, resulting in moderate to high degree of heterogeneity. A more recent meta-analysis of 15 RCTs that enrolled patients with T2DM, CKD, and/or HF found no significant effect of SGLT2i on the incidence of amputation and fracture with no heterogeneity (90). This meta-analysis revealed a consistent increased risk of DKA as well as an increased risk of volume depletion. Although the meta-analysis included RCTs that enrolled patients with CKD, SGLT2i showed superiority but not inferiority in reducing the risk of acute kidney injury. These findings indicate the need for adequate patient education, including alerting patients to subjective symptoms of DKA and dehydration, such as lightheadedness, fatigue, abdominal pain, nausea, and vomiting, to confer a greater efficacy of SGLT2i treatment.

## Discussion

In this review, we have summarized the results of RCTs and real-world data for SGLT2i treatment in patients with T2DM, HF, and CKD. Although the primary action of SGLT2i involves inhibition of SGLT2 expressed on proximal tubule cells, these drugs exhibit pleiotropic effects, which include reductions in body weight, blood pressure, intra-glomerular pressure, hyperuricemia, inflammation and oxidative stress, inhibition of the sympathetic nervous system, and improvements in erythropoiesis, cardiac energy metabolism and vascular function (91–93). The reduction in body weight is at least partly explained by a reduction in fat mass (94, 95). SGLT2i also reduce epicardial fat that releases pro-inflammatory mediators (96, 97). In addition, dapagliflozin was reported to inhibit the NLRP3 inflammasome, resulting in attenuation of fibrosis in diabetic mice (98). Further, the benefits on the heart may be mediated by improving myocardial energy efficiency. Ketone bodies were also elevated in patients treated with SGLT2i, which might contribute to improved cardiac function (99, 100). The increase in the erythropoietin level achieved by SGLT2i treatment appears to involve the suppression of hepcidin and ferritin and an increase of transferrin receptor protein 1 (101–103), which would correct anemia and improve clinical outcomes (104). Furthermore, inhibition of SGLT2 reduces the ATP-dependent tubular workload and oxygen requirements, alleviating hypoxia (105). Mitochondrial dysfunction has been implicated in both HF and CKD (106, 107), and several possible mechanisms were proposed by which SGLT2i preserve normal mitochondrial function (93). These effects probably contribute collectively to the cardiorenal protective effects of SGLT2i. Notably, an improvement in cardiac function leads to an improvement in renal function, and vice versa, owing to the mutual interactions between the heart and the kidney. Moreover, SGLT2i seem to prevent the onset of T2DM in patients with HF or CKD (108, 109), emphasizing the importance of using this drug in patients with CRM syndrome.

Although the efficacy of SGLT2i in CRM interactions is becoming increasingly clear, some issues remain unclear. Acute HF management with SGLT2i is one such example, and has received growing attention. A relatively small RCT was conducted and reported a significant clinical benefit of SGLT2i on the prognosis and symptoms in patients hospitalized for acute HF (110). A large-scale RCT is ongoing (NCT04363697) and is expected to provide rigorous evidence regarding this aspect. The efficacy and safety of SGLT2i in populations excluded from previous RCTs, such as the super-elderly (age >85 years) and/or highly frail individuals are also needed. Because of the considerable time and effort required to conduct such RCTs, it will be helpful to obtain evidence using real-world data to address the remaining issues and further solidify the CRM protective effects of SGLT2i.

Considering the emerging concept of CRM interactions, SGLT2i have shown better performance than initially expected. SGLT2i have not only been used as an antidiabetic agent but have also become a new treatment option for patients with HF (irrespective of LVEF or care settings) and/or CKD. Regardless of the presence of diabetes, patient management focusing on cardiorenal protection is important in terms of prognosis and QOL. SGLT2i can contribute to better treatment strategies for a huge number of patients suffering from CRM diseases.

## Author contributions

All authors contributed to the conception of the review. HA and AN wrote the first draft, and NM and TY revised it critically for important intellectual content. All authors contributed to the article and approved the submitted version.
